# Giant gluteal and vesical plexiform neurofibromas in a patient with neurofibromatosis type 1: a case report

**DOI:** 10.1186/s13256-023-04315-z

**Published:** 2024-01-13

**Authors:** Imen Sassi, Mohamed Amine Bouida, Anis Hasnaoui, Ines Zemni, Tarek Ben Dhieb

**Affiliations:** 1 Surgical Oncology Department, Salah Azaiez Institute of Oncology, Faculty of Medicine of Tunis, Tunis El Manar University, Tunis, Tunisia; 2 Department of General Surgery, Menzel Bourguiba Hospital, Faculty of Medicine of Tunis, Tunis El Manar University, 7050 Menzel Bourguiba, Bizerta, Tunisia

**Keywords:** Neurofibromatosis 1, Plexiform neurofibroma, Hydronephrosis, MEK inhibitor, Case report

## Abstract

**Background:**

Neurofibromatosis type 1 is a neurocutaneous genetic disorder caused by mutations in the NF1 gene, resulting in the formation of benign tumors called neurofibromas. The most common type of tumor seen in patients with neurofibromatosis type 1 is the slow-growing and benign neurofibroma, with a subtype called plexiform neurofibroma being particularly common and causing pain, functional impairment, and cosmetic disfigurement.

**Case presentation:**

We report the case of a 20-year-old North African female patient with a history of neurofibromatosis type 1 who presented with a growing mass in her right gluteal region, which was later diagnosed as a giant cutaneous neurofibroma. Imaging studies revealed infiltration in several regions, including the urinary bladder wall, resulting in significant bilateral hydronephrosis. The patient is currently being monitored, and no excisional procedures are planned.

**Conclusions:**

Neurofibromatosis type 1 can cause a variety of clinical symptoms, including the development of large plexiform neurofibromas. It is important to closely monitor patients with neurofibromatosis type 1 for the early detection of neurofibromas. Early detection and prompt surgical intervention are essential for preventing complications.

## Background

Neurofibromatosis type 1 (NF1), also known as von Recklinghausen disease, is a neurocutaneous genetic disorder with an estimated worldwide incidence of 1 in 4950 individuals [1, 2]. The disorder arises from mutations in the NF1 gene, which codes for the protein neurofibromin, a regulator of RAS-MAPK signalling and tumor suppressor. The loss of neurofibromin function leads to dysregulation of cell growth and proliferation, ultimately forming benign tumors known as neurofibromas, which can arise from any peripheral nerve [3].

Among the types of tumors seen in patients with NF1, neurofibromas are the most common and typically grow slowly and remain benign [4]. A subtype of neurofibroma called a plexiform neurofibroma (PN) is particularly common in patients with NF1 and is characterized by diffuse involvement of multiple nerves, which can cause pain, functional impairment, and cosmetic disfigurement [5].

We report the case of a patient with NF1 who developed a giant plexiform neurofibroma in the gluteal region, with accompanying infiltration of the urinary bladder wall causing bilateral hydronephrosis.

## Case presentation

**Fig. 1:**
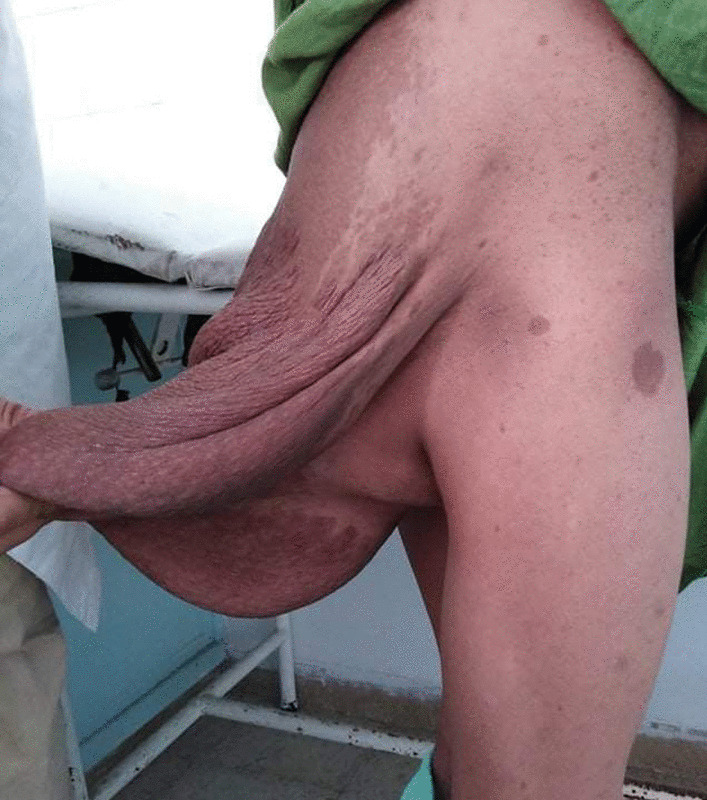
Multiple café au lait spots with a plexiform neurofibroma of the sacral region appearing darker in color compared with the surrounding skin, measuring 30 × 25 cm

A 20-year-old North African female patient, born to consanguineous parents, presented with a medical history of Hirschsprung’s disease and a concurrent diagnosis of NF1 with a growing mass in the left gluteal region that had been present since childhood but increased in size over the last few years. Physical examination revealed multiple café au lait spots and neurofibromas of different sizes on the limbs and trunk. A giant cutaneous neurofibroma measuring 30 × 25 cm of the sacral region was also observed (Fig. 1).

**Fig. 2:**
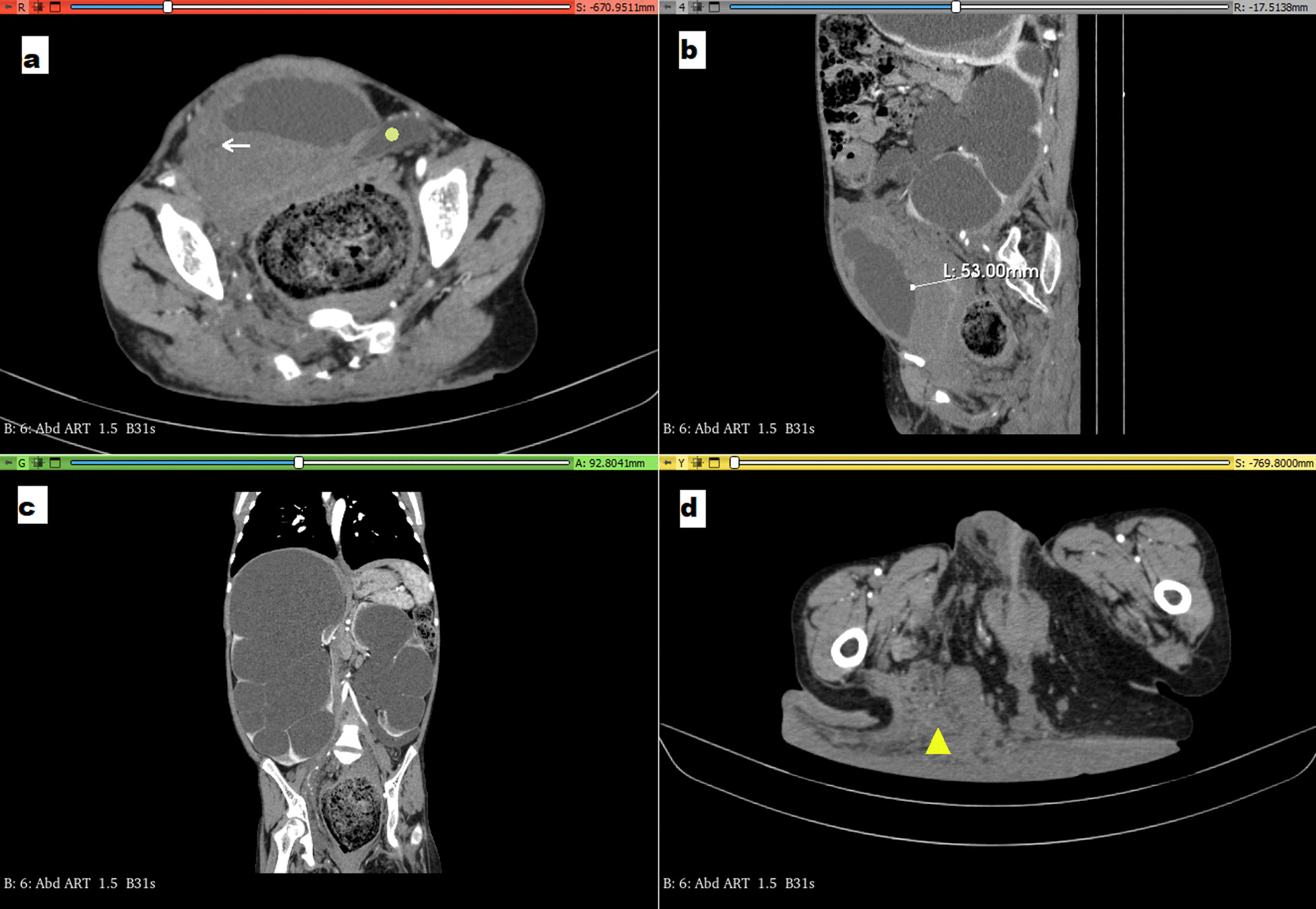
**a** Axial contrast-enhanced computed tomography (CT) image revealing the thickened wall of the urinary bladder with stenosis of the right ureter at the vesicoureteral junction (arrow) and the dilated left ureter (circle) just before joining the bladder **b** Sagittal CT image showing infiltration of the wall of the urinary bladder (53 mm thick) **c** Coronal CT image showing bilateral hydronephrosis **d** Axial CT image revealing the infiltration of neurofibromatosis in the right and left gluteal regions with dermal–hypodermal invasion (triangle)

Computed tomography (CT) scan revealed nodular infiltration of neurofibromatosis in the right and left gluteal regions with dermal–hypodermal invasion (Fig. 2d) and poorly defined infiltrating nodules in the pelvic region. The pelvic organs, including the uterus, ovaries, rectal wall, and bladder wall (Fig. 2b) were infiltrated. The thickened bladder wall caused the stenosis of the vesicoureteral junctions (Fig. 2a) and consequently significant bilateral hydronephrosis (Fig. 2c). The patient was referred to urology, but no endoscopic procedure could be performed due to massive infiltration. The patient is currently being followed up with stable radiological lesions and normal renal function. No excisional procedures are planned.

## Discussion and conclusions

PNs are pathognomonic for NF1 and can result in significant disfigurement and functional limitations. This type of neurofibroma typically arises in early childhood and becomes more apparent as a cutaneous lesion once it has fully developed [6]. PNs can vary greatly in size and location, with many developing along large nerve trunks or regions with increased adipose tissue deposition [5]. These tumors can cause the affected nerve to thicken and expand, leading to hypertrophy of surrounding tissues and possible compression of neighboring structures [7].

PNs can involve not only superficial tissues, but also deep tissues or internal organs, such as the gastrointestinal tract or urogenital area, without any apparent external extension [5]. A higher incidence in female patients during adolescence was noted for internal neurofibromas [8].

Imaging studies are valuable for assessing the location and extent of lesions. Ultrasonography (US) is a valuable diagnostic tool in differentiating between benign and malignant lesions. However, its accuracy may be insufficient, and hence it is commonly used in combination with magnetic resonance imaging (MRI). MRI is the gold standard for preoperative assessment. T1-weighted images of neurofibromas (NFs) typically depict a low-to-intermediate signal, while T2-weighted images show a high signal [9]. The “target sign,” which is a homogeneous hyperintense region, is a characteristic pattern commonly observed in NFs [10, 11].

Computed tomography (CT) imaging is an effective diagnostic modality for identifying nodular, fusiform, or cluster-like lesions that have a lower density than muscle (20–30 UH). This lower density is attributed to lipid inclusions present in Schwann cells, adipocytes, cystic degeneration, and myxoid stroma. The enhancement pattern of these lesions after contrast injection varies, with some showing homogeneous or heterogeneous enhancement. In our case, the patient was found to have extensive involvement of the cervical, abdominal, and pelvic structures with the invasion of adjacent tissues, as identified on the CT scan.

To reduce the risk of potential complications, such as malignant transformation into malignant peripheral nerve sheath tumors (MPNSTs), which pose a significant concern, with an estimated lifetime risk of 15.8% of PNs transforming into MPNSTs, it is essential to closely monitor PNs [12].

The management of plexiform neurofibromas can be classified into three main approaches: conservative, surgical, and medical therapies. Conservative management involves regular monitoring, pain control, and psychological support. Various studies have suggested monitoring the cases of PN with CT or MRI every 6 months to 1 year [13]. Surgical resection is effective for excluding malignancy and is usually only used in certain cases, such as large superficial lesions and craniofacial lesions [5]. However, surgery, especially for diffuse plexiform neurofibromas, is challenging because of the risk of massive bleeding due to tumor spread and tissue invasion. Medical therapy with mitogen-activated protein kinase kinase (MEK) inhibitors, especially selumetinib, is a promising option for inoperable and symptomatic PN, with positive results in pediatric trials [5]. Sawaragi *et al*. have shown that a carefully selected group of patients with extensive disfiguring plexiform neurofibromas with pain and/or threat to function may benefit from MEK inhibitors, either as monotherapy or in combination with surgery [5]. Other centers have demonstrated the use of MEK inhibitors to reduce tumor size to enable surgical excision [14]. However, it is crucial to note that while selumetinib has been approved by the Food and Drug Administration (FDA) in the USA and has recently been authorized in the United Kingdom [15], access to MEK inhibitors is yet to be authorized in our country. Nevertheless, given the extensive and diffuse nature of the lesions, and the associated surgical challenges, we strongly believe that our patient would be an excellent candidate for selumetinib therapy.

PNs can cause a variety of symptoms depending on their location, including visual problems, respiratory difficulties, motor impairments, etc. These symptoms are primarily due to the tumor’s direct pressure on surrounding tissues and can become life-threatening when vital organs are compressed. The most common symptoms are pain and motor deficits. The presence of infection and chronic disease can worsen the prognosis of this serious condition [16]. In a pediatric cohort study assessing the mortality and morbidity profiles associated with NF1, it was observed that children afflicted with symptomatic neurofibromas and NF1 exhibited an elevated mortality rate of 3.2% [17]. Notably, the size of the tumor plays a pivotal role in determining the severity of the clinical manifestations, with larger tumors correlating with more pronounced pathological effects. These functional impairments exert a detrimental influence on the overall quality of life [18], underscoring the essential need for ongoing surveillance to assess the clinical impact of therapeutic interventions.

In our clinical case, we are committed to the rigorous monitoring of our patient, vigilantly screening for signs of tumor growth, potential complications, or the ominous transformation into MPNST. This monitoring regimen involves the routine utilization of computed tomography (CT) or magnetic resonance imaging (MRI) scans at intervals ranging from 6 months to 1 year.

NF1 is a rare genetic disorder that can present with a wide range of clinical manifestations. Giant plexiform neurofibromas are a rare but significant complication of NF1. Early detection and prompt surgical intervention can prevent complications and improve outcomes. It is crucial to closely monitor patients with NF1 to detect the development of neurofibromas as early as possible, including the use of imaging studies to identify the presence of tumors. The optimal management of these tumors remains unclear. Therefore, decisions regarding the management of NF1 should be made in specialized centers and be the subject of a multicenter study to ensure adequate patient care.

## Data Availability

This work was created with the utmost respect for the code of ethics under the supervision of the Medical and Ethics Committee of the Salah Azaiez Institute of Oncology.

## Abbreviations

NF1: Neurofibromatosis type 1
PN: Plexiform neurofibroma
CT: Computed tomography
US: Ultrasonography
MPNSTs: Malignant peripheral nerve sheath tumors
